# Machine learning-based prediction model and web calculator for postoperative LDVT in colorectal cancer

**DOI:** 10.3389/fonc.2025.1673705

**Published:** 2025-10-10

**Authors:** Zhihui Zhang, ShiCong Xu, MeiXuan Song, WeiRong Huang, ManLin Yan, XianRong Li

**Affiliations:** ^1^ School of Nursing, Southwest Medical University, Luzhou, Sichuan, China; ^2^ Department of Gastrointestinal Surgery, The Affiliated Hospital of Southwest Medical University, Luzhou, Sichuan, China; ^3^ Nursing Department, Ya’an People’s Hospital, Ya’an, Sichuan, China

**Keywords:** machine learning, predictive model, colorectal cancer, lower limb deep vein thrombosis, risk assessment

## Abstract

**Background:**

Lower limb deep vein thrombosis (LDVT) is a common but often underdiagnosed complication after colorectal cancer (CRC) surgery. Its early symptoms are subtle, and delayed detection can lead to post-thrombotic syndrome or even life-threatening events. However, effective tools for early risk assessment are lacking.

**Objective:**

To identify risk factors for postoperative LDVT in CRC patients and develop a machine learning (ML)-based risk prediction model with an accessible web calculator.

**Methods:**

This retrospective study included 1,200 CRC patients undergoing radical surgery. A modeling cohort of 1,000 patients (January 2021–December 2022) was randomly split 8:2 into training and testing sets, and 200 patients (March–August 2024) formed an external validation cohort. Risk factors were screened using univariate analysis and least absolute shrinkage and selection operator (LASSO) regression. Eight ML models were constructed and compared based on area under the curve (AUC), accuracy, sensitivity, and F1-score. The best-performing model was interpreted using SHapley Additive exPlanations (SHAP), and a web-based calculator was developed.

**Results:**

Among 1,200 patients, 369 (30.75%) developed LDVT (31.5% in the modeling cohort, 27% in the validation cohort). Seventeen variables were associated with LDVT in univariate and LASSO analyses, and the top 10 were used to build models. The random forest (RF) model showed the best performance, with AUCs of 0.942, 0.897, and 0.891 in the training, testing, and validation sets, respectively, demonstrating high accuracy and generalizability. SHAP analysis identified D-dimer, preoperative intestinal obstruction, Caprini score, age, intraoperative blood loss, and diabetes as major predictors, with D-dimer having the strongest impact. A web-based calculator (https://crc-ldvt.shinyapps.io/RF-model/) was constructed to provide individualized risk estimation.

**Conclusion:**

This study developed and validated a robust ML-based model for predicting postoperative LDVT in CRC patients. The RF model, incorporating key clinical predictors, demonstrated high predictive performance and clinical relevance. The online calculator enables rapid, individualized risk assessment and may help guide early prevention strategies, reducing postoperative complications and improving patient outcomes.

## 1 Introduction

According to the Global Cancer Statistics (GLOBOCAN 2022), there were 1.926 million new cases of colorectal cancer (CRC) and 904,000 deaths worldwide in 2022 (1).The incidence and mortality rates of CRC ranked third and second, respectively, among all malignancies (2, 3). By 2030, the global burden of colorectal cancer is projected to increase by approximately 60%, posing a severe threat to human health (4, 5). The imaging and clinical diagnostic incidence of venous thromboembolism (VTE) after colorectal cancer surgery can be as high as 40%, with pulmonary embolism (PE) accounting for approximately 5% (6). Lower limb deep vein thrombosis (DVT), particularly in the mid-to-distal veins, is more common and typically manifests as localized pain and gait disturbances (7). The consequences of VTE are profound, including prolonged hospitalization, delayed cancer treatment, the development of post-thrombotic syndrome, and even death, significantly increasing medical expenses (8). Moreover, studies indicate that thrombus formation may also promote tumor growth and metastasis, raising the mortality rate of cancer patients to 9.2%, second only to cancer progression itself (9, 10). However, only 50% of patients clinically present with obvious symptoms such as lower limb swelling and localized deep tenderness (11). This indicates that most cases of venous thromboembolism (VTE) are asymptomatic in the early stages due to partial obstruction of the venous lumen by thrombi or compensatory function of superficial veins, making early detection challenging. As a result, in patients with a low risk of lower limb deep vein thrombosis (LDVT), the potential harms of thromboprophylaxis may outweigh its benefits. Therefore, an ideal LDVT prevention strategy should be based on risk stratification, accurately identifying high-risk individuals and implementing targeted preventive measures. The National Comprehensive Cancer Network (NCCN) guidelines recommend using high-quality risk assessment tools to screen high-risk patients and develop effective stratified prevention strategies accordingly to reduce the incidence of LDVT (12).

However, existing predictive models for postoperative lower limb deep vein thrombosis (LDVT) in colorectal cancer patients predominantly rely on traditional logistic regression methods (13). These models emphasize testing causal hypotheses and selecting models based on goodness-of-fit within the data. However, the strict linear assumptions inherent in both approaches make it challenging to capture nonlinear relationships in large, complex datasets (14, 15). Additionally, these models primarily depend on static variables for evaluation, lacking the capability for dynamic prediction and thus struggling to adapt to the complexity of postoperative changes in patient conditions (16). Machine learning algorithms, as a branch of artificial intelligence, operate at the intersection of computer science and statistical methodologies (17). They can integrate diverse data sources and provide accurate predictions. The application of machine learning techniques in the medical field has brought significant advancements in disease diagnosis and prevention. In recent years, machine learning has been widely utilized for risk prediction in various clinical conditions, such as postpartum stress urinary incontinence (18), disability in the elderly (19), and obesity in children (20). Moreover, machine learning has played a significant role in drug development and personalized medicine (21, 22). With the increasing richness of comprehensive patient information in electronic health records, including examination and diagnostic data, coupled with the rapid advancements in machine learning technology, new opportunities have emerged for the development of high-performance predictive models.

**Figure 1:**
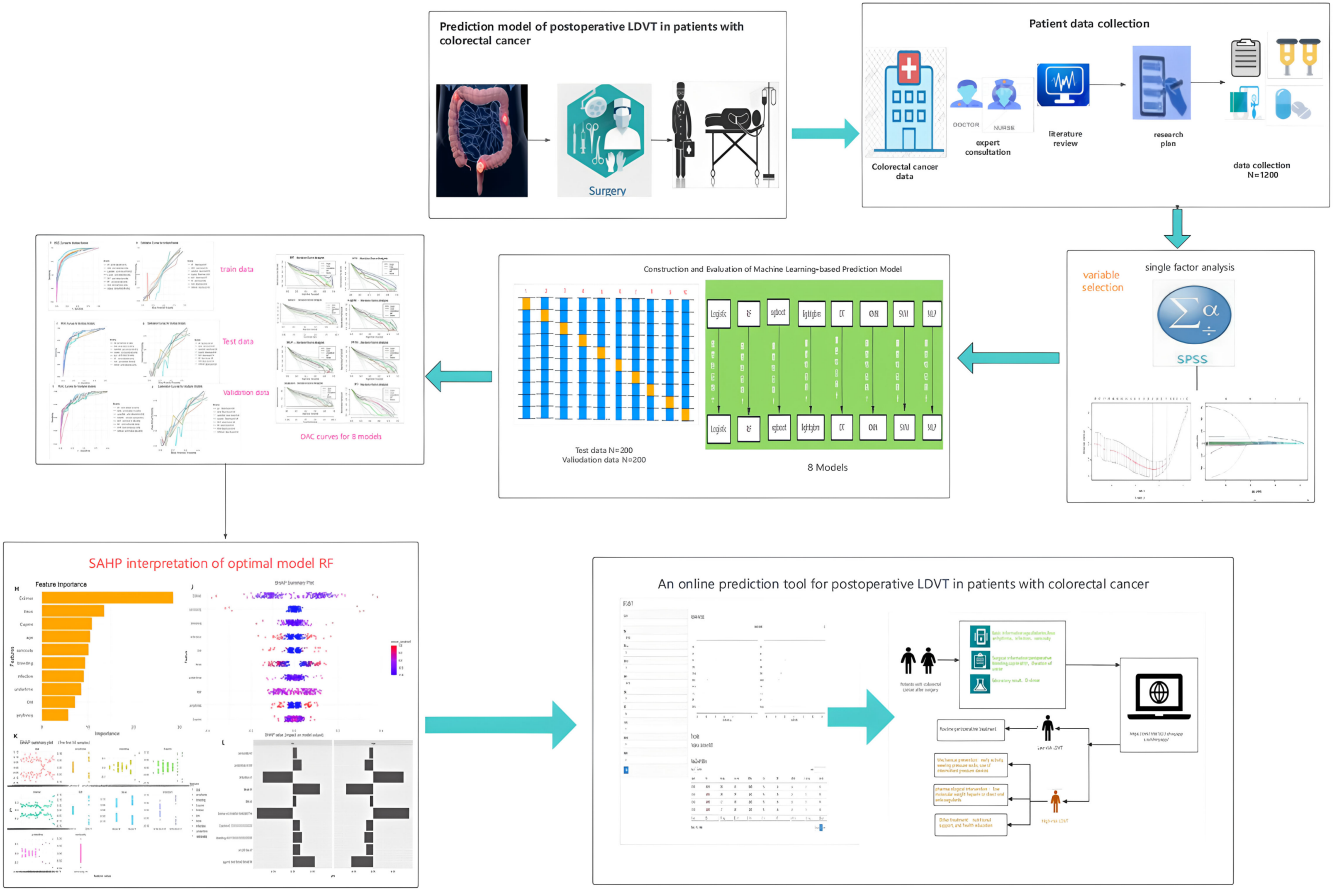
Summary diagram.

Therefore, this study aims to construct a predictive model for lower limb deep vein thrombosis (LDVT) complications following colorectal cancer surgery using machine learning algorithms. The research will incorporate a wider range of more effective predictive factors to analyze the patterns and relationships between various features and LDVT, ultimately providing a personalized and precise predictive model applicable in clinical settings. An overview of the study design and findings is provided in the summary diagram (**Figure 1**).

## 2 Methods

### 2.1 Study design and population selection

This study is a retrospective cohort study that collected data from 1,000 patients who underwent radical colorectal cancer surgery between January 2021 and December 2022 for model development, and data from 200 patients collected between March and August 2024 for external validation (**Supplementary Figure S1**). Inclusion criteria were (1): diagnosis of stage I–III colorectal cancer according to the Chinese Guidelines for Diagnosis and Treatment of Colorectal Cancer (2020 edition), confirmed by imaging and pathology (2); receipt of radical colorectal cancer surgery (3); no evidence of lower limb deep vein thrombosis before surgery; and (4) bilateral lower limb color Doppler ultrasound screening within two weeks postoperatively to detect both symptomatic and asymptomatic deep vein thrombosis. Exclusion criteria included (1): presence of severe chronic diseases or major organ failure (2); Treat patients who were discharged prematurely; and (3) missing key data ≥ 20%. This study complied with the Declaration of Helsinki and was approved by the hospital ethics committee (approval number: KY2023420).

### 2.2 Research variable

Based on clinical expertise and previous research evidence (**Supplementary Table S1**), the variables included demographic characteristics (age, sex, smoking, and alcohol consumption), physical measurements (BMI), medical history (hypertension and diabetes), surgical factors (intraoperative blood loss and anesthesia duration), and the first postoperative laboratory test results (D-dimer, white blood cell count, neutrophil count, and other related biomarkers).

### 2.3 Definitions and results

According to the standard terminology definitions provided by the World Health Organization (WHO) and the Centers for Disease Control and Prevention (CDC), lower extremity deep vein thrombosis (LDVT) refers to the abnormal formation of thrombi within the deep venous system of the lower limbs—such as the popliteal, femoral, or iliac veins—resulting in partial or complete obstruction of the vessel lumen.

In this study, LDVT was defined as the occurrence of lower extremity deep vein thrombosis within two weeks after colorectal cancer surgery, including both symptomatic and asymptomatic cases, all of which were confirmed by imaging examinations.

### 2.4 Data preprocessing

To improve modeling efficiency and data quality, data preprocessing was performed prior to model development. Binary variables were encoded as 0 and 1, unordered categorical variables were one-hot encoded, and ordinal variables were labeled starting from 0. Numerical variables were normalized to the [0,1] range to minimize the impact of scale differences. Variables with minimal missing data were imputed using various methods (e.g., Amelia in R 4.4.1, mice, or the mi package), while variables with ≥20% missing values were excluded. Remaining missing values were handled via multiple imputation(MI). Outliers were identified using boxplots and replaced with the mean or median according to the data distribution.

### 2.5 Feature selection

During feature selection, univariate analysis was first performed on the training set to identify variables potentially associated with lower-limb deep vein thrombosis (LDVT) after colorectal cancer surgery, thereby eliminating clearly irrelevant features. Subsequently, the variables that passed this screening were further refined using least absolute shrinkage and selection operator (LASSO) regression in R software (version 4.4.1). By introducing L1 regularization, LASSO effectively addresses multicollinearity among variables, with the optimal regularization parameter determined through 10-fold cross-validation, selecting the lambda value within one standard error of the minimum (lambda.1se). Finally, the top 10 variables ranked by feature importance across different machine learning models were selected as the final input features, aiming to balance model complexity and predictive performance, reduce overfitting risk, and enhance the generalizability and clinical utility of the model.

### 2.6 Model construction and validation

The modeling cohort was randomly divided into a training set (80%) and an internal test set (20%), while an independent cohort collected between March and August 2024 served as the external validation set. The test set and external validation set were used solely for model performance evaluation and did not participate in any model training, feature selection, or hyperparameter optimization, to avoid data leakage and ensure independent and robust model evaluation. All model development steps were conducted using the training set. Hyperparameters were optimized through grid search combined with 10-fold cross-validation to enhance generalizability and minimize overfitting risk. Specifically, the training set was split into 10 subsets; in each iteration, 9 subsets were used for training and 1 subset for validation, repeating this process 10 times. The average validation metrics were then used to evaluate model performance (23, 24). Grid search systematically explored different hyperparameter combinations within a predefined range, selecting the configuration that achieved the best validation results (**Supplementary Table S2**). A total of eight machine learning prediction models were constructed: logistic regression (LR), random forest (RF), support vector machine (SVM), decision tree (DT), XGBoost, LightGBM, multilayer perceptron (MLP), and k-nearest neighbors (KNN).

After model training, predictive performance was evaluated on both the internal test set and the external validation set. Evaluation metrics included the area under the ROC curve (AUC), accuracy, sensitivity (recall), specificity, positive predictive value (PPV), negative predictive value (NPV), F1-score, Youden’s index (J_index), Brier score, and balanced accuracy. A multidimensional comparison was performed to comprehensively assess the strengths and weaknesses of each model.

### 2.7 Model interpretation

Interpreting machine learning models, especially complex “black box” models, can be challenging. The Shapley Additive Explanation (SHAP) method, grounded in game theory, addresses this challenge by ranking the importance of input features and quantifying their contributions to the model’s predictions (25). SHAP can calculate both positive and negative contributions of each feature, providing local explanations (for individual samples) as well as global explanations (for overall feature importance), thereby enhancing model transparency and clinical interpretability. In this study, interpretability analysis was conducted using the shap package in R.

### 2.8 Web calculator

To support clinical application, the final prediction model was deployed on a Shiny-based web platform. This online application allows clinicians to input relevant patient variables and obtain an individualized probability of LDVT occurrence, assisting in postoperative risk assessment and decision-making.

### 2.9 Statistic analysis

Descriptive statistics and group comparisons were performed using R version 4.4.1. Categorical data were expressed as frequencies and percentages (%) and compared using the chi-square test. Continuous data with a normal distribution were presented as mean ± standard deviation (Mean ± SD) and compared using independent-samples t-tests or analysis of variance (ANOVA). Non-normally distributed data were expressed as median and interquartile range [Median (IQR)] and analyzed with the Mann-Whitney U test. Multiple categorical variables were compared using ANOVA. A significance level of P < 0.05 was considered statistically significant, and all tests were two-sided.

## 3 Results

### 3.1 Univariate analysis

This study included a total of 1,200 patients who underwent colorectal cancer surgery. Based on the occurrence of lower limb deep vein thrombosis (LDVT) after surgery, patients were divided into a non-LDVT group (831 cases, mean age 61.96 years) and an LDVT group (369 cases, mean age 68.48 years). The overall incidence of LDVT was 30.75%. The missing rates of variables ranged from 0.00% to 5.25%, with the highest missing rate observed in tumor staging (5.25%). The incidence of LDVT in the modeling group (n = 1,000) and the external validation group (n = 200) was 31.5% and 27%, respectively. Univariate analysis in the training set (n = 800) showed that 40 variables, including age, preoperative intestinal obstruction, surgical approach, Caprini score, blood type, and anesthesia time, were significantly associated with LDVT occurrence (P < 0.05). In contrast, 23 variables, such as pathological type, body mass index (BMI), total protein, lipoproteins, and red blood cell count, showed no significant association (P > 0.05) (**Table 1**).

**Table 1:** Univariate analysis of relevant variables (Training Group, n=800).

Variable	No occurrence (n=541)	Occurrence (n=259)	*z/t/*χ^2^	*P*
Age, mean (SD),years	61.68 ± 11.44	68.89 ± 9.88	-9.162	<0.001
Gender, (n %)			0.465	0.495
Female	214(39.56)	109(42.08)		
Male	421(61.46)	188(59.68)		
BMI, mean(SD),kg/m^2^	22.36 ± 3.18	22.73 ± 3.47	-1.462	0.144
Smoking History, mean(SD),years	7.33 ± 13.75	10.67 ± 16.94	-2.768	<0.001
Alcohol Consumption History, mean(SD),years	4.22 ± 10.99	7.55 ± 15.07	-3.175	0.002
Blood type, (n %)			12.631	0.006
A	162(30.17)	100(38.76)		
AB	45(8.38)	15(5.81)		
B	125(23.28)	72(27.91)		
O	205(38.18)	71(27.52)		
History of abdominal surgery, (n %)	118(21.81)	76(29.34)	5.409	0.020
History of lower limb surgery, (n %)	28(5.18)	33(12.74)	14.234	<0.001
Lower limb varicosities, (n %)	8(1.48)	12(4.63)	7.150	0.007
Diabetes, (n %)	46(8.50)	56(21.62)	27.097	<0.001
Chronic pulmonary disease, (n %)	102(18.85)	96(37.07)	31.191	<0.001
Coronary heart disease, (n %)	18(3.33)	34(13.13)	27.679	<0.001
Arrhythmia, (n %)	51(9.43)	83(32.05)	64.264	<0.001
Hypertension, (n %)			16.293	0.001
Grade I	64(11.83)	35(13.51)		
Grade II	44(8.13)	40(15.44)		
Grade III	16(2.96)	15(5.79)		
Intestinal obstruction, (n %)	117(21.63)	158(61.00)	120.389	<0.001
Hemorrhagic/tarry stool, (n %)	308(57.04)	168(64.86)	4.454	0.035
Preoperative Length of Stay, mean (SD),days	8.80 ± 5.71	9.10 ± 7.23	-0.648	0.517
Number of Catheters on the First Postoperative Day, mean (SD),count	2.84 ± 0.79	3.07 ± 0.85	-3.721	<0.001
Anesthesia Duration, median(IQR),min	230(187.5,270)	245 (200,300)	-3.634	<0.001
Intraoperative blood loss, median(IQR),ml	20 (20,50)	30(20,50)	-5.985	<0.001
Urinary Catheter Duration, mean(SD),days	4.45 ± 1.89	5.93 ± 3.06	-7.127	<0.001
Nasogastric Tube Duration, mean(SD),days	0.72 ± 2.06	1.77 ± 3.41	-4.557	<0.001
Duration in ICU, mean(SD),days	0.23 ± 1.61	1.00 ± 3.78	-3.148	0.002
Caprini, mean(SD)	4.76 ± 2.23	6.35 ± 2.61	-8.443	<0.001
Surgical Method, (n %)			30.880	<0.001
Laparoscopic	529(97.78)	229(88.42)		
Open Surgery	12(2.22)	30(11.58)		
Intraoperative Position, (n %)			23.471	<0.001
Modified Lithotomy Position	397(73.38)	193(74.52)		
Scissors Position	130(24.03)	41(15.83)		
Supine Position	14(2.59)	25(9.65)		
Blood Transfusion	43(7.95)	43(16.60)	13.672	<0.001
Placement of Nasogastric Tube, (n %)	83(15.34)	82(31.66)	28.489	<0.001
Postoperative ICU Admission, (n %)	24(4.44)	53(20.46)	51.721	<0.001
Vascular invasion, (n %)	126(23.42)	70(27.24)	1.364	0.243
Electrolyte disorder, (n %)	47(8.69)	50(19.31)	18.531	<0.001
Clean enema, (n %)	519(95.93)	241(93.05)	3.065	0.080
Postoperative infection, (n %)	60(11.09)	90(34.75)	64.351	<0.001
Postoperative stoma, (n %)	147(27.17)	115(44.40)	23.608	<0.001
Central venous catheterization, (n %)	10(1.85)	13(5.02)	6.307	0.012
Anastomotic leak, (n %)	11(2.03)	11(4.25)	3.210	0.073
Chemotherapy, (n %)	162(29.94)	75(28.96)	0.082	0.775
Wound fat liquefaction, (n %)	11(2.03)	4(1.54)	0.228	0.633
WBC, mean(SD),10^9^/L	9.47 ± 3.36	9.69 ± 3.20	-0.903	0.367
NEW, mean(SD),10^9^/L	8.19 ± 3.92	8.47 ± 3.40	-0.983	0.326
LYM, mean(SD),10^9^/L	1.66 ± 3.87	1.82 ± 3.63	-0.556	0.579
MCHC, mean(SD), g/L	319.80 ± 23.25	317.14 ± 31.15	1.350	0.177
PLT, median(IQR),10^9/^L	202.50(159,247)	193(156,254)	-1.228	0.219
FIB, mean(SD),g/L	3.84 ± 1.24	4.24 ± 1.38	-3.759	<0.001
PT, mean(SD), s	11.84 ± 1.14	12.60 ± 1.73	-6.383	<0.001
D-dimer, mean(SD),mg/L	0.82 ± 0.95	2.45 ± 2.43	-10.238	<0.001
APTT, mean(SD),s	27.68 ± 3.94	28.81 ± 4.90	-3.242	0.001
TP, median(IQR),g/L	54.70(51,57.9)	54.30(50,59)	-0.763	0.445
ALB, mean(SD),g/L	32.72 ± 12.25	32.41 ± 16.80	0.300	0.764
PAB, mean(SD),g/L	138.06 ± 43.71	127.71 ± 45.75	3.087	0.002
TC, mean(SD),mmol/L	3.75 ± 1.01	3.46 ± 1.05	3.785	<0.001
TG, mean(SD),mmol/L	1.17 ± 1.11	1.36 ± 0.87	-2.350	0.019
HDL, mean(SD),mmol/L	1.24 ± 5.92	0.91 ± 0.31	0.901	0.368
LDL, mean(SD),mmol/L	2.24 ± 0.75	2.02 ± 0.79	3.829	<0.001
GLU, mean(SD),mmol/L	6.17 ± 1.90	6.80 ± 2.44	-3.658	<0.001
CEA, median(IQR),ng/mL	4.67(2.8,9.5)	6.16(3.3,16.1)	-3.600	<0.001
RBC, mean(SD),10¹²/L	3.86 ± 0.63	3.83 ± 0.67	0.671	0.502
HGB, mean(SD),g/L	112.23 ± 27.83	109.69 ± 21.06	1.302	0.193
Pathological Type, (n %)			0.510	0.775
colon	226(41.77)	115(44.40)		
Colorectal	6(1.11)	3(1.16)		
Rectum	309(57.12)	141(54.44)		
T(n %)			4.403	0.354
T0	5(0.95)	2(0.80)		
T1	31(5.86)	16(6.37)		
T2	93(17.58)	41(16.33)		
T3	334(63.14)	147(58.57)		
T4	66(12.48)	45(17.93)		
N(n %)			2.969	0.397
N0	303(57.50)	136(54.40)		
N1	139(26.38)	77(30.80)		
N2	82(15.56)	37(14.80)		
N3	3(0.57)	0(0.00)		
M(n %)			8.859	0.031
M0	480(91.78)	214(86.29)		
M1	41(7.84)	33(13.31)		
M3	0(0.00)	1(0.40)		
M4	2(0.38)	0(0.00)		
Tumor Stage, (n %)			7.889	0.162
0	25(4.89)	12(5.00)		
I	102(19.96)	53(22.08)		
II	182(35.62)	74(30.83)		
III	163(31.90)	71(29.58)		
IV	38(7.44)	27(11.25)		

BMI, Body Mass Index; WBC, White Blood Cell Count; LYM, Lymphocyte Count; MCHC, Mean Corpuscular Hemoglobin Concentration; PLT, Platelet Count; FIB, Fibrinogen; PT, Prothrombin Time; D-dimer, D-dimer; APTT, Activated Partial Thromboplastin Time; TP, Total Protein; ALB, Albumin; PAB, Prealbumin; TC, Total Cholesterol; TG, Triglycerides; HDL, High-Density Lipoprotein Cholesterol; LDL, Low-Density Lipoprotein Cholesterol; GLU, Fasting Blood Glucose; CEA, Carcinoembryonic Antigen; RBC, Red Blood Cell Count; HGB, Hemoglobin; T, Primary Tumor; N, Lymph Node Metastasis; M, Distant Metastasis; A, Blood Type A; B, Blood Type B; O, Blood Type O; AB, Blood Type AB; Number of Catheters on the First Postoperative Day (urinary catheters, abdominal drains, nasogastric tubes, and rectal tubes).

### 3.2 LASSO regression

**Figure 2:**
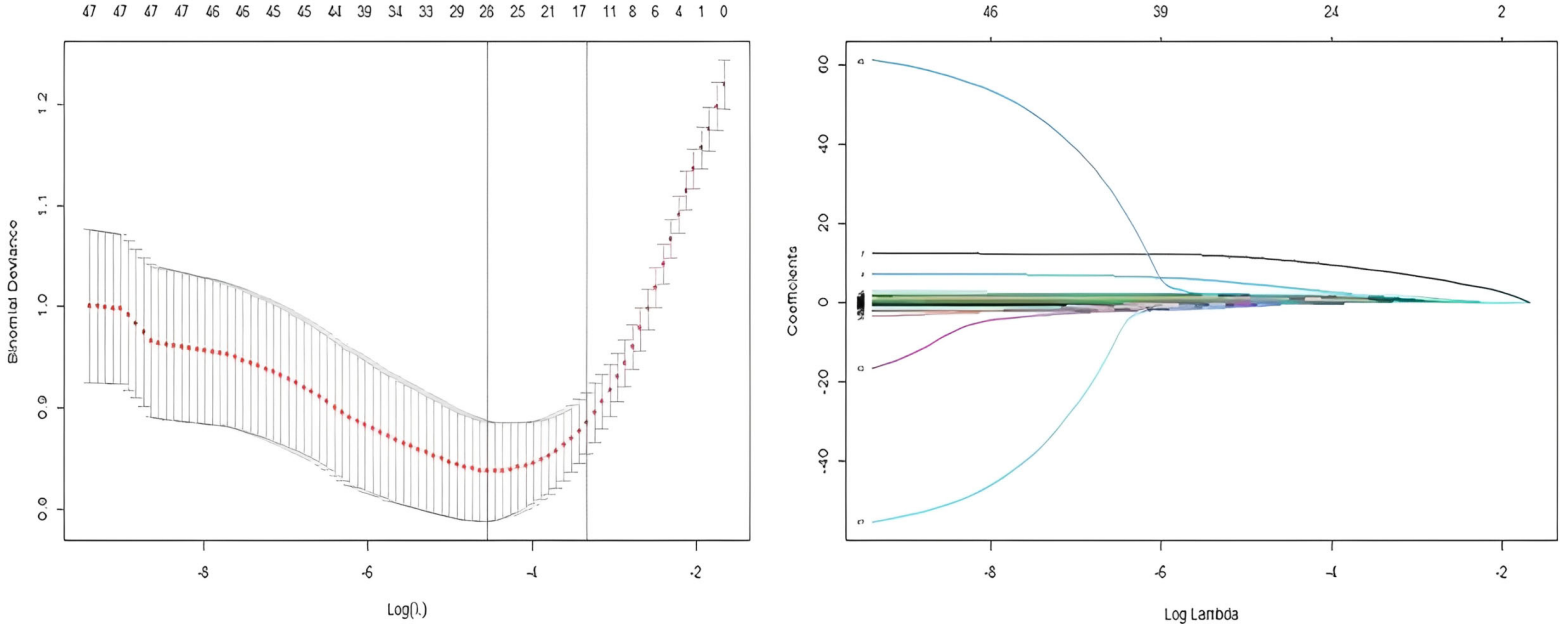
Combined visualization of LASSO regression: variable selection process and coefficient path plot.

In this study, 40 variables initially screened by univariate analysis from the modeling group were further selected using LASSO regression in R, with 10-fold cross-validation applied via the cv.glmnet function to identify the optimal penalty parameter λ. Variables with non-zero coefficients under λ1se were retained, yielding 17 final predictors (**Figure 2**, **Table 2**).

**Table 2:** Supplement LASSO results of 34 variables after univariate analysis.

Variable	Regression coefficient
Caprini	0.51542535
age	1.71653609
Number of catheters on the first postoperative day	0.80158728
Intraoperative blood loss	1.25979734
Time of catheter placement	1.64598754
D-dimer	8.89615263
Alcohol consumption history	0.09183638
Nasogastric Tube Duration	0.11293929
Hematochezia/tarry stools	0.14269731
Intestinal obstruction	0.67667691
Postoperative stoma	0.01772579
History of lower limb surgery	0.09732719
Diabetes	0.30682158
infection	0.47752239
Lower limb varicosity	0.73267884
Coronary heart disease	0.05514663
Arrhythmia	0.74030500

### 3.3 Baseline comparison of training set, internal validation set, and external validation set

Based on the 17 variables selected by the LASSO regression method, the baseline characteristics of the training set (n=800), the test set (n=200), and the external validation set (n=200) were compared (**Table 3**). The results indicated that certain baseline differences existed among the three groups, mainly between the external validation set and the modeling datasets (training and test sets). This was expected due to differences in the time periods and populations from which the data were collected. Subsequent model evaluations were performed on strictly separated test and external validation sets to ensure the robustness and generalizability of the results.

**Table 3:** Comparison of clinical features of the training set, test set and external validation set.

Variable	Training set (n=800)	Internal validation set (*n* = 200)	External validation set (*n* = 200)	*F/H*	*P*
age	63.89 ± 11.59	63.88 ± 11.20	64.31 ± 10.36	0.117	0.889
Coronary heart disease	49(6.13)	17(8.50)	13(6.50)	1.470	0.479
Arrhythmia	125(15.63)	30(15.00)	49(24.50)	9.612	0.008
History of lower limb surgery	52(6.50)	15(7.50)	8(4.00)	2.347	0.309
Diabetes	113(14.12)	21(10.50)	22(11.00)	139.035	<0.001
Lower limb varicosity	27(3.38)	8(4.00)	3(1.50)	2.378	0.305
Alcohol consumption history	5.32 ± 12.67	5.76 ± 12.36	2.04 ± 2.92	7.177	0.001
Inserting a gastric tube	155(19.38)	49(24.50)	30(15.00)	5.773	0.078
Hematochezia/tarry stools	463(57.88)	122(61.00)	105(52.50)	3.095	0.213
Intestinal obstruction	286(35.75)	61(30.50)	67(33.50)	2.058	0.357
Postoperative stoma	277(34.63)	58(29.00)	57(28.50)	4.196	0.123
postoperative infection	138(17.25)	34(17.00)	28(14.00)	1.236	0.539
Number of catheters on the first postoperative day	2.91 ± 0.80	3.04 ± 0.79	2.67 ± 0.87	10.685	<0.001
Intraoperative blood loss	30(20,50)	30(20,50)	30(20,40)	0.979	0.613
Ureteral catheter duration	4.98 ± 2.57	4.88 ± 2.24	4.59 ± 2.31	1.955	0.142
D-dimer	1.35 ± 1.80	1.35 ± 1.45	1.53 ± 1.34	1.008	0.365
Caprini score	5.29 ± 2.57	4.88 ± 2.66	6.89 ± 2.06	39.861	<0.001

### 3.4 Model construction

**Figure 3:**
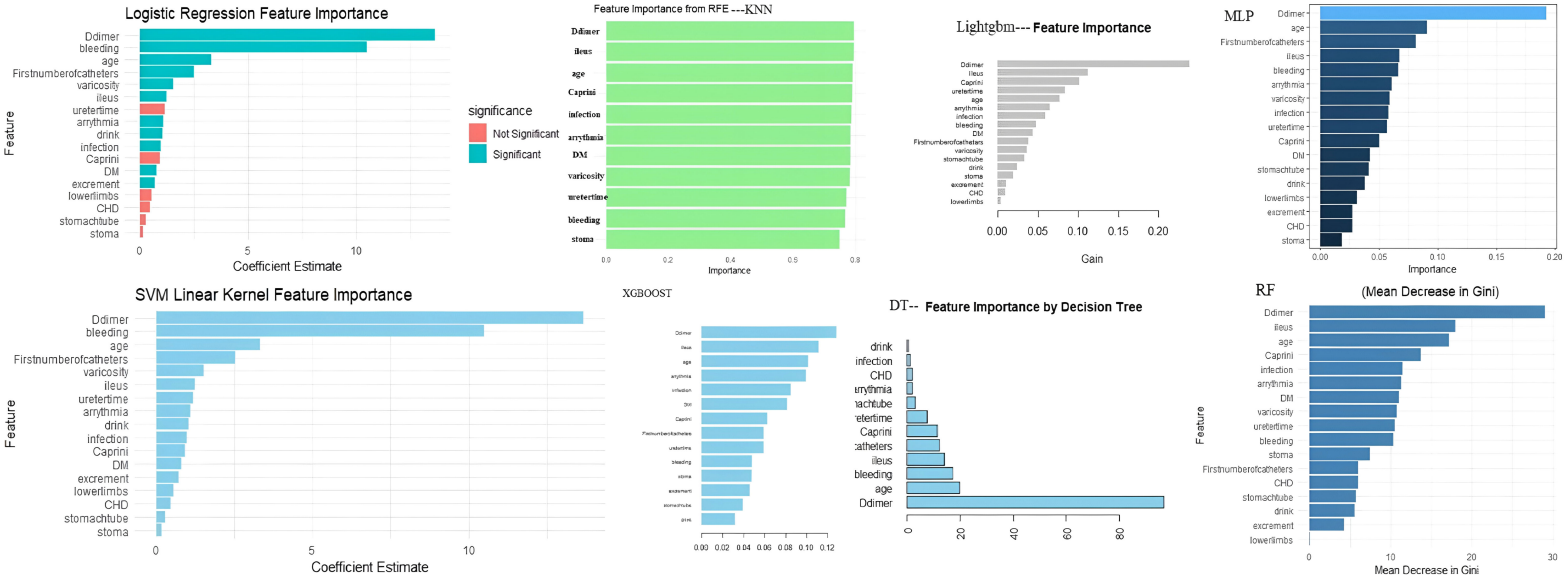
Important features of eight models.

In this study, we first performed univariate analysis on the training set and identified 40 potentially influential variables out of a total of 63 independent variables. To further refine and determine the core variables for modeling, LASSO regression analysis was applied to these 40 variables, with the optimal λ at the 1-SE criterion selected based on the training set, ultimately identifying 17 key variables. Next, these 17 variables were evaluated for feature importance using eight different algorithms, including logistic regression, random forest, support vector machine, decision tree, XGBoost, LightGBM, multilayer perceptron, and K-nearest neighbors. Based on the characteristics of each model, we ranked the variables by importance. We also tested models including more variables (e.g., the top 8, 9, 11, and 13 variables) and found that although the AUC in the training set slightly increased, the stability in the validation set did not improve significantly. In some cases, the Brier Score even increased slightly, suggesting that including additional variables may introduce redundant information and reduce generalizability. Therefore, we ultimately selected the top 10 variables from each model for model construction. (**Figure 3**, **Supplementary Tables S3**, **S4**).

### 3.5 Model performance

#### 3.5.1 Performance evaluation of eight models on the training set

**Figure 4:**
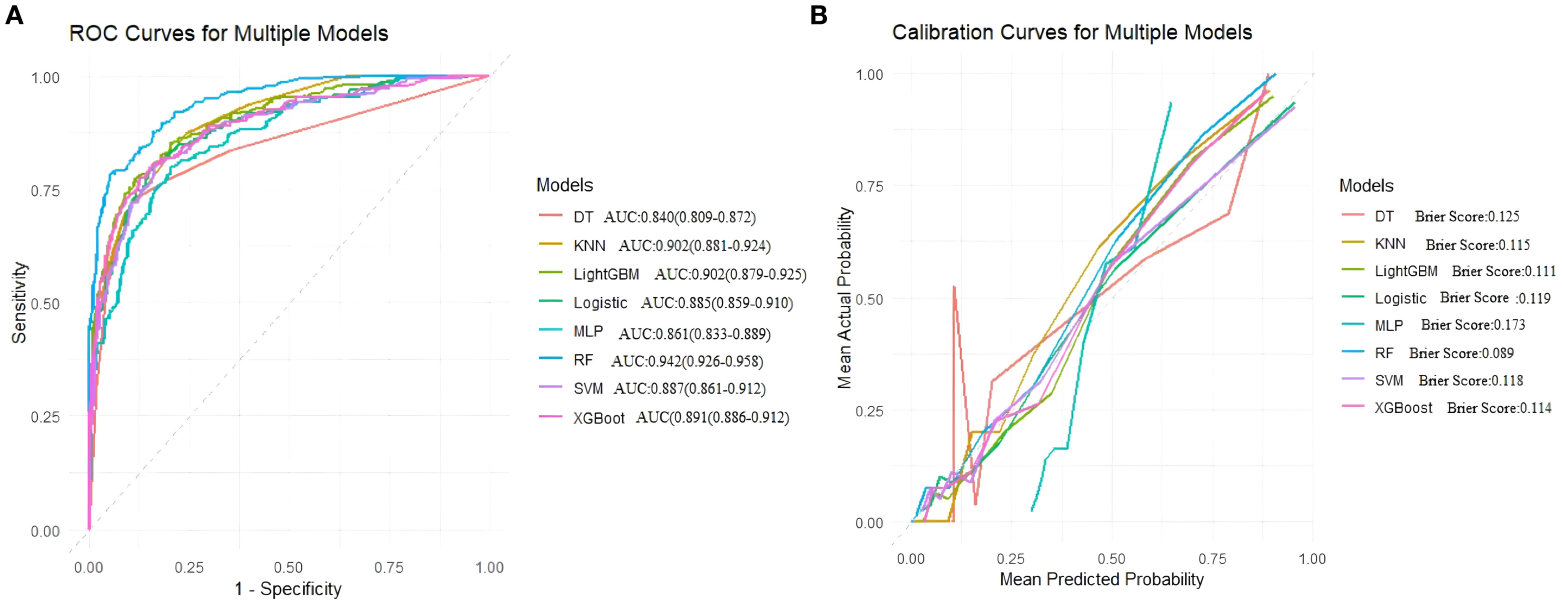
**(A)** Receiver operating characteristic (ROC) curves for models, **(B)** Calibration curves for the same models. The Brier score is presented for models.

On the training set, the Random Forest (RF) model showed the best overall performance, with an AUC of 0.942 (95% CI: 0.926–0.958), accuracy of 0.894, and F1-score of 0.924. It achieved high sensitivity (0.945) and balanced accuracy (0.864), with a low Brier Score (0.089). LightGBM and XGBoost also performed well (AUCs 0.902 and 0.891), while SVM and Logistic Regression showed solid but slightly weaker results (AUCs 0.887 and 0.885). Decision Tree, KNN, and MLP had lower overall performance. Overall, RF was the most effective model on the training data. (**Table 4**, **Figure 4**).

**Table 4:** Supplement performance evaluation of eight models on training set.

Model	AUC(95%CI)	Accuracy	Sensitivity	Specificity	PPV	NPV	F1_score	J_index	Brier score	Balanced accuracy
Logistic	0.885(0.859-0.910)	0.828	0.838	0.806	0.904	0.695	0.869	0.643	0.119	0.822
SVM	0.887(0.861-0.912)	0.826	0.832	0.813	0.907	0.690	0.868	0.646	0.118	0.823
RF	0.942(0.926-0.958)	0.894	0.945	0.782	0.904	0.868	0.924	0.727	0.089	0.864
XGBoost	0.891(0.866-0.912)	0.839	0.865	0.782	0.896	0.727	0.880	0.647	0.114	0.823
LightGBM	0.902(0.879-0.925)	0.850	0.885	0.774	0.895	0.756	0.890	0.659	0.111	0.829
MLP	0.861(0.833-0.889)	0.796	0.796	0.798	0.895	0.642	0.843	0.593	0.173	0.797
KNN	0.902(0.881-0.924)	0.794	0.757	0.873	0.928	0.623	0.834	0.630	0.115	0.815
DT	0.840(0.809-0.872)	0.836	0.889	0.722	0.874	0.749	0.881	0.611	0.125	0.805

#### 3.5.2 Internal validation performance evaluation of eight models

**Figure 5:**
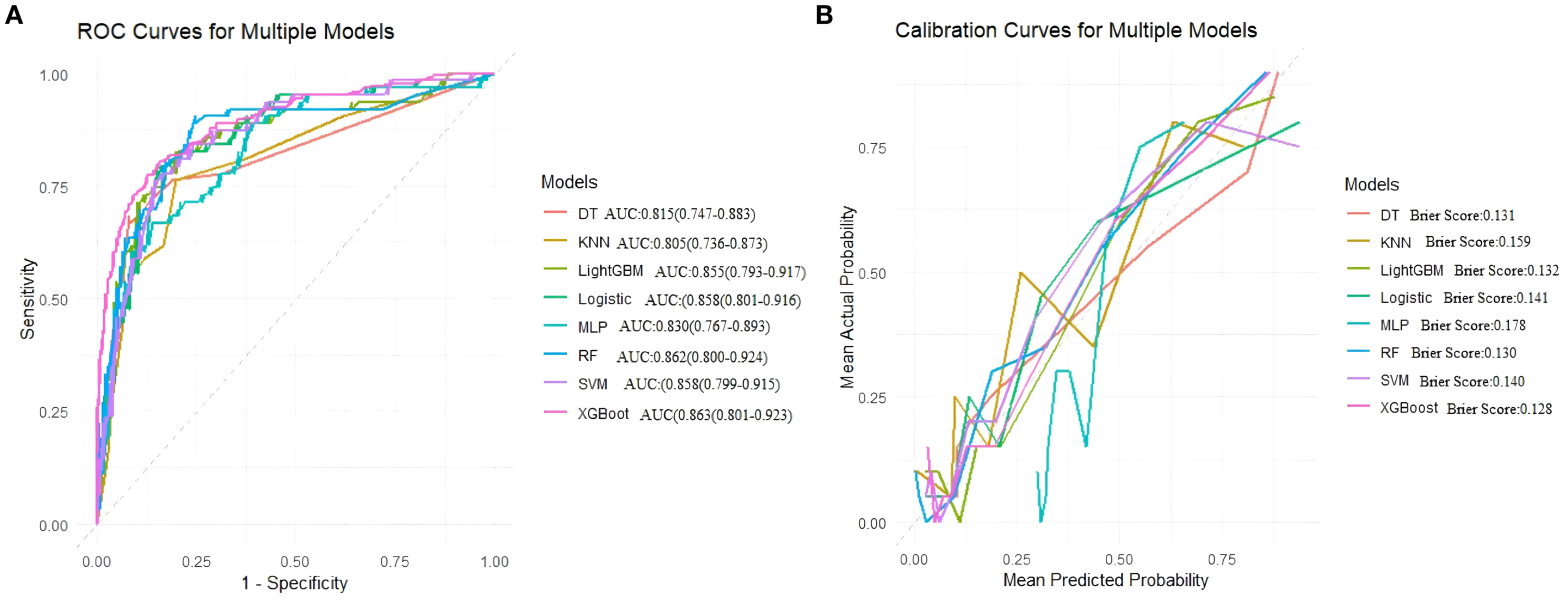
Area under the ROC curve and Brier score curve for the internal validation set. **(A)** Receiver operating characteristic (ROC) curves for models, **(B)** Calibration curves for the same models. The Brier score is presented for models.

In internal validation, the random forest (RF) model performed excellently, achieving an AUC of 0.862, sensitivity of 0.905, accuracy of 0.820, and an F1 score of 0.873. XGBoost showed a comparable AUC of 0.863, but overall had a slightly lower recall than RF. LightGBM, support vector machine (SVM), and logistic regression also performed well but did not surpass RF. Decision tree, k-nearest neighbors (KNN), and multilayer perceptron (MLP) models had lower AUC values and generally weaker overall metrics. RF was the most effective model on the internal validation data (**Table 5**, **Figure 5**).

**Table 5:** Supplement performance evaluation of eight models on internal validation test set.

Model/indicator	AUC(95%CI)	Accuracy	Sensitivity	Specificity	PPV	NPV	F1_score	J_index	Brier score	Balanced accuracy
Logistic	0.858(0.801-0.916)	0.825	0.847	0.778	0.892	0.700	0.869	0.624	0.141	0.812
SVM	0.858(0.799-0.915)	0.815	0.832	0.778	0.891	0.681	0.860	0.610	0.140	0.805
RF	0.862(0.800-0.924)	0.820	0.905	0.635	0.844	0.755	0.873	0.540	0.130	0.770
XGBoost	0.863(0.801-0.923)	0.825	0.847	0.778	0.829	0.700	0.869	0.624	0.128	0.812
LightGBM	0.855(0.793-0.917)	0.820	0.861	0.730	0.874	0.708	0.868	0.591	0.132	0.796
MLP	0.830(0.767-0.893)	0.760	0.788	0.698	0.850	0.603	0.818	0.487	0.178	0.743
KNN	0.805(0.736-0.873)	0.79	0.803	0.762	0.88	0.64	0.84	0.565	0.159	0.782
DT	0.815(0.747-0.883)	0.825	0.891	0.683	0.859	0.741	0.875	0.573	0.131	0.787

#### 3.5.3 Performance evaluation of eight models on external validation set

**Figure 6:**
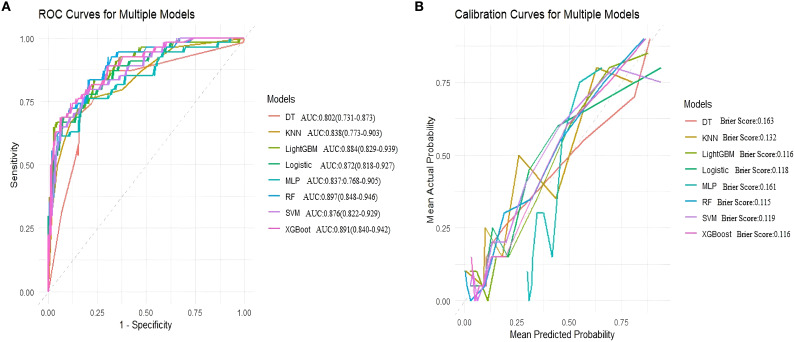
Area under the ROC curve and Brier score curve for the external validation set. **(A)** Receiver operating characteristic (ROC) curves for models, **(B)** Calibration curves for the same models. The Brier score is presented for models.

In external validation, Random Forest (RF) performed best with an AUC of 0.897, accuracy of 0.805, balanced sensitivity (0.815) and specificity (0.778), and low error (Brier Score 0.115). XGBoost and SVM also showed good results but slightly less balanced. LightGBM, Logistic Regression, and MLP had moderate performance. Decision Tree and KNN performed poorly. Overall, RF was the top model. (**Table 6**, **Figure 6**).

**Table 6:** Supplement performance evaluation of eight models on external validation set.

Model/indicator	AUC(95%CI)	Accuracy	Sensitivity	Specificity	PPV	NPV	F1_s core	J_index	Brier score	Balanced accuracy
Logistic	0.872(0.818-0.927)	0.790	0.808	0.741	0.894	0.588	0.849	0.549	0.118	0.774
SVM	0.876(0.822-0.929)	0.790	0.801	0.759	0.900	0.586	0.848	0.561	0.119	0.780
RF	0.897(0.848-0.946)	0.805	0.815	0.778	0.908	0.609	0.859	0.593	0.115	0.796
XGBoost	0.891(0.840-0.942)	0.790	0.781	0.815	0.919	0.579	0.844	0.596	0.116	0.798
LightGBM	0.884(0.829-0.939)	0.790	0.815	0.722	0.888	0.591	0.850	0.537	0.116	0.769
MLP	0.837(0.768-0.905)	0.800	0.842	0.685	0.879	0.617	0.860	0.528	0.161	0.764
KNN	0.838(0.773-0.903)	0.670	0.623	0.796	0.892	0.439	0.734	0.420	0.132	0.710
DT	0.802(0.731-0.873)	0.800	0.836	0.704	0.884	0.613	0.859	0.539	0.163	0.770

#### 3.5.4 Decision curve analysis

**Figure 7:**
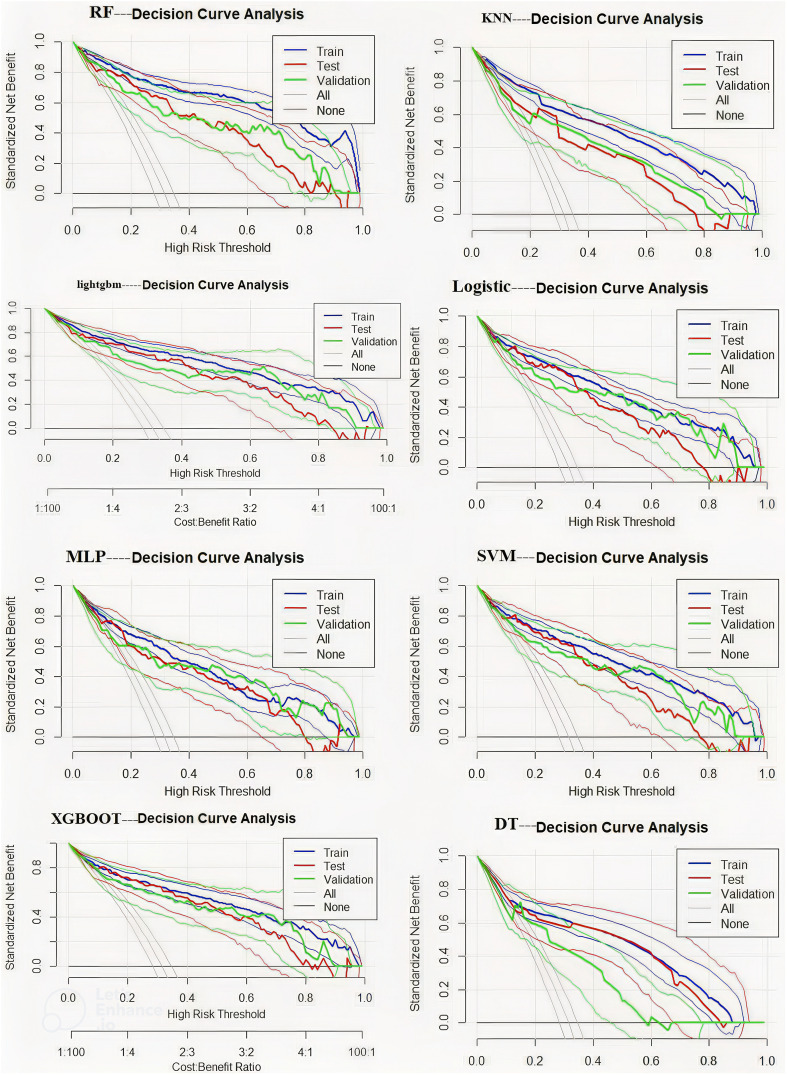
Decision curve analysis plot.

This study compared eight machine learning models for predicting postoperative DVT using decision curve analysis. The RF model demonstrated favorable net benefits across different risk thresholds, particularly within the range of 0.2–0.5, where the net benefit remained relatively stable and was clearly superior to other strategies. XGBoost and LightGBM performed well at lower risk levels. Logistic Regression was stable but less accurate. SVM and MLP had limited use, especially at high risk. KNN and Decision Tree performed worst. RF is recommended as the best model (**Figure 7**).

### 3.6 Model interpretation

**Figure 8:**
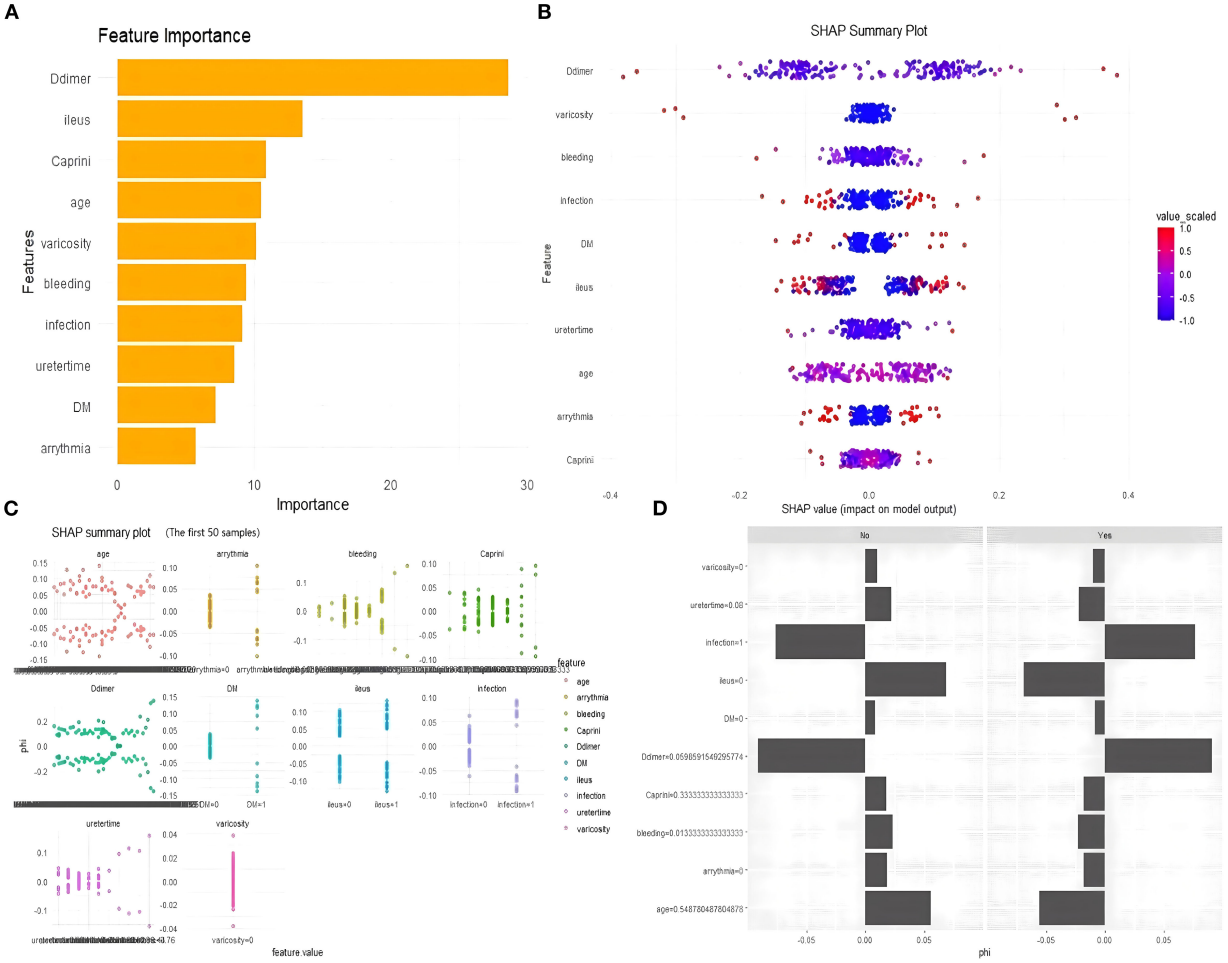
SHAP explanation plot. **(A)** Feature importance plot. **(B)** SHAP summary plot. **(C)** Individual explanation plot. **(D)** The top 50 patients, SHAP values.

The feature importance plot (**Figure 8A**) highlights D-dimer as the most influential predictor in the RF model, aligning with its established role in thrombosis. Other key features, including preoperative intestinal obstruction, Caprini score, and age, also showed considerable importance for clinical reference. The SHAP summary plot (**Figure 8B**) further revealed that elevated D-dimer, along with varicose veins, intraoperative bleeding, infection, diabetes, and intestinal obstruction, substantially increased LDVT risk. The individual explanation plot (**Figure 8C**) demonstrated how these features contributed to a specific patient’s risk, with high D-dimer, diabetes, and infection raising risk, while younger age, absence of varicose veins, and lower blood loss were protective. Across the top 50 patients, SHAP values (**Figure 8D**) illustrated the impact of age, arrhythmia, postoperative bleeding, and Caprini score on predictions, with positive SHAP values indicating higher risk and negative values suggesting lower risk. Overall, these results emphasize how SHAP enhances individualized risk assessment and supports clinical decision-making for postoperative LDVT.

**Figure 9:**
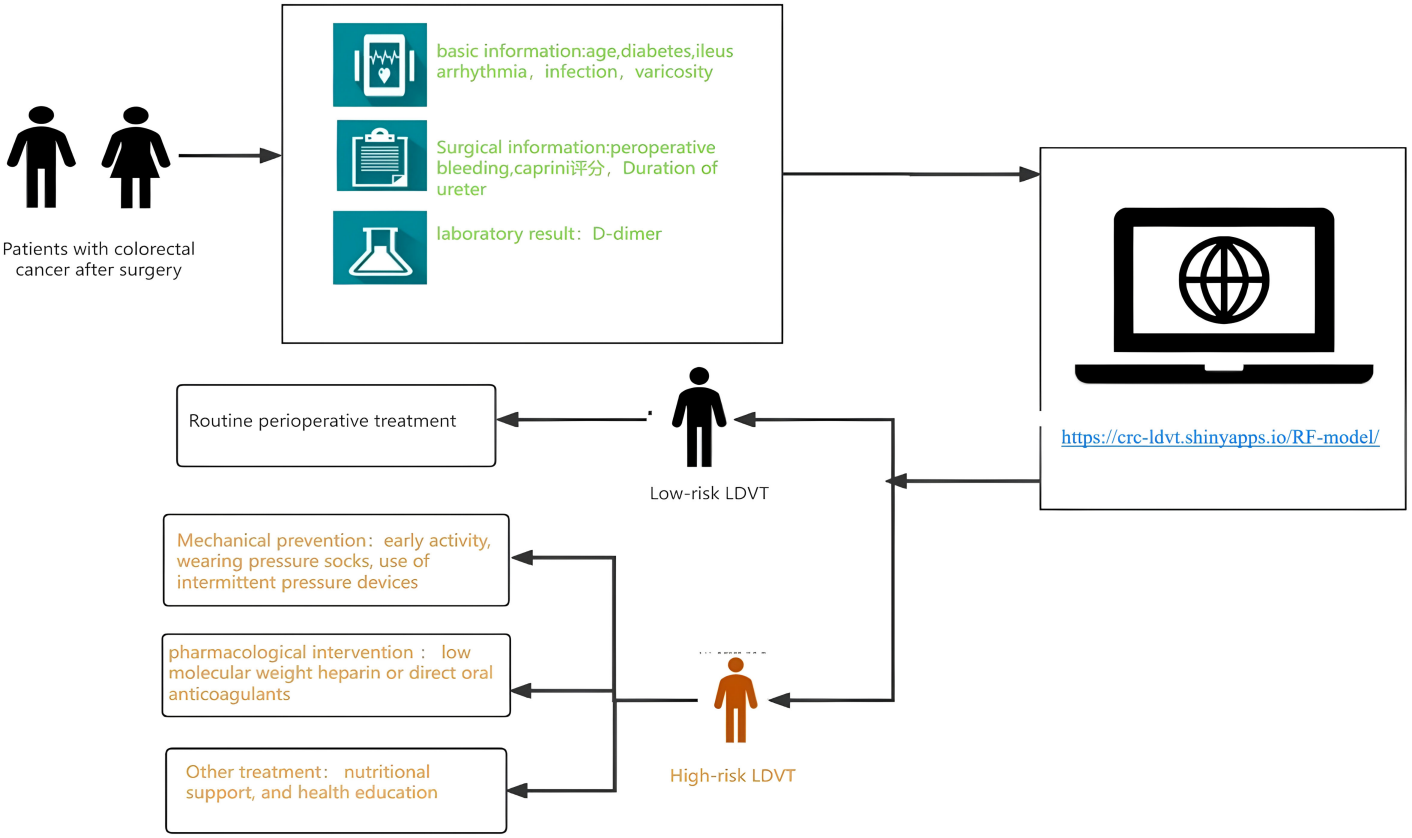
Workflow of the web-based LDVT risk prediction tool for patients with colorectal cancer after surgery. The model integrates patients’ basic, surgical, and laboratory information to estimate LDVT risk through an online calculator (https://crcldvt.shinyapps.io/RF-model). Based on the predicted risk, patients are stratified into low- and high-risk groups, receiving routine or intensified preventive interventions accordingly.

### 3.7 Usage process of the online tool

Based on the random forest (RF) algorithm, we developed an online risk prediction tool for postoperative lower extremity deep vein thrombosis (LDVT) in patients with colorectal cancer (https://crc-ldvt.shinyapps.io/RF-model/) to identify high-risk individuals. Medical staff can use this tool to predict LDVT risk, with the workflow illustrated in **Figure 9**. By entering key clinical variables, such as age, Caprini score, D-dimer levels, and bleeding time, users can quickly obtain individualized risk probabilities. The interface also visually displays the contribution of each variable to the model’s prediction, using Mean Decrease Accuracy and Mean Decrease Gini to reflect the relative importance of each predictor. A table at the bottom presents detailed data for multiple observed cases, including the input variables and corresponding predicted outcomes, facilitating comparison and analysis.This tool not only provides precise, individualized risk assessment to support clinical decision-making but also clearly illustrates variable importance. When the predicted LDVT risk is low, patients may receive standard postoperative management; when the predicted risk is high, medical staff can provide increased attention and implement comprehensive interventions tailored for high-risk patients. These interventions include mechanical prophylaxis (e.g., early mobilization, compression stockings, intermittent pneumatic compression), pharmacological interventions (e.g., low-molecular-weight heparin or direct oral anticoagulants), nutritional support, and patient education. Moreover, by dynamically monitoring patient status and balancing thromboprophylaxis with bleeding risk during anticoagulant therapy, the tool can help reduce the incidence of thrombosis and related complications, promote postoperative recovery, and improve patients’ quality of life.

## 4 Discussion

Lower limb deep vein thrombosis (LDVT) often develops insidiously during the early postoperative period in patients undergoing colorectal cancer surgery. Therefore, timely risk stratification and targeted prevention within the first two weeks after surgery are essential to reduce complications and improve recovery. In this study, we initially identified 40 candidate variables through univariate analysis and further optimized them using LASSO regression, ultimately selecting 17 core predictors. Based on feature importance rankings, eight machine learning (ML) models were developed using the top 10 features from each algorithm. Among these, the random forest (RF) model demonstrated the best predictive performance. Feature importance analysis consistently highlighted D-dimer, preoperative bowel obstruction, age, Caprini score, intraoperative blood loss, and varicose veins as the most influential predictors for LDVT. SHAP-based interpretability further revealed how these clinical variables impact LDVT risk at the individual level, breaking the so-called “black box” of ML models and enhancing their clinical applicability in early postoperative settings.

This study employed machine learning methods to develop a predictive model for lower limb deep vein thrombosis (LDVT) within two weeks following colorectal cancer surgery. Among the evaluated variables, D-dimer consistently ranked as the most important feature across all algorithms, highlighting its stable and prominent role in thrombosis risk prediction. These findings not only reinforce the clinical value of D-dimer from a data-driven perspective but also provide indirect evidence supporting its central role in the underlying pathophysiology of LDVT.

Mechanistically, D-dimer is a specific degradation product of cross-linked fibrin generated during fibrinolysis. Its elevation reflects simultaneous activation of coagulation and fibrinolytic pathways, typically indicating an ongoing process of thrombus formation and breakdown (26, 27). In the postoperative setting, surgical trauma, tissue injury, inflammation, venous stasis, and a hypercoagulable state collectively contribute to this process, thereby increasing circulating D-dimer levels (28). Unlike traditional scoring systems such as the Caprini score, D-dimer offers the advantage of temporal sensitivity, capturing an individual’s thrombotic risk status at a specific point in time. This dynamic nature may explain its superior predictive performance in our models compared to static variables. It not only aids in identifying the presence of thrombosis but also assists in assessing the rate of progression, therapeutic response, and recurrence risk.

Moreover, D-dimer is a routinely available, cost-effective laboratory test with excellent clinical applicability. In the context of postoperative management, a key challenge lies in balancing the prevention of LDVT with the risk of excessive bleeding caused by anticoagulation. D-dimer serves as a pivotal tool in this risk-benefit trade-off by enabling real-time risk stratification and treatment adjustment. Dynamic monitoring of D-dimer levels can thus inform individualized anticoagulation strategies, facilitating optimal outcomes through precise thromboprophylaxis and timely intervention.

This study identified preoperative bowel obstruction as a high-importance predictor for LDVT across all machine learning models, suggesting it may be an underrecognized yet clinically significant risk factor. Mechanistically, bowel obstruction may contribute to thrombosis through increased intra-abdominal pressure, venous stasis, dehydration, and systemic inflammation—all of which create a hypercoagulable state and impair venous return.

As a severe gastrointestinal complication, bowel obstruction not only increases surgical risk but also promotes thrombogenesis via multiple pathways. Intestinal distension can compress the iliac and femoral veins, reducing blood flow velocity (29). Concurrently, vomiting, reduced oral intake, and fluid shifts may lead to hemoconcentration and increased blood viscosity (7, 30). Inflammatory responses further exacerbate the prothrombotic state by releasing cytokines (e.g., IL-6, TNF-α), which damage the endothelium, activate coagulation, and enhance platelet aggregation (31). Future studies are needed to clarify whether the severity or duration of obstruction correlates with thrombosis risk in a dose-dependent manner.

Age, intraoperative blood loss, and the Caprini score showed consistent importance in this study and are supported by well-established pathophysiological mechanisms. Advancing age is associated with vascular aging, endothelial dysfunction, and venous valve insufficiency—all of which contribute to impaired venous return and increased stasis (32). Moreover, elderly individuals often have higher blood viscosity and reduced mobility, further elevating thrombosis risk (33–35). Excessive intraoperative blood loss may lead to hypoperfusion, hemodynamic instability, and activation of intrinsic coagulation pathways, thereby promoting thrombus formation (36). Although the Caprini score is widely used for perioperative thrombosis risk stratification, it relies heavily on static clinical features and lacks intraoperative variables such as bowel obstruction and blood loss, which were identified as strong predictors in our model. Integrating such surgery-specific factors may enhance its predictive accuracy in real-world settings.

Other variables, including infection, prolonged urinary catheterization, arrhythmia, diabetes, and varicose veins, demonstrated moderate yet biologically plausible predictive value in selected models. These factors may exert greater influence in specific subgroups. For instance, prolonged catheter use is linked to immobility and venous stasis (37); infection induces systemic inflammation and hypercoagulability (38); arrhythmia alters hemodynamic stability (39); and diabetes contributes to endothelial dysfunction (40, 41). Although these features may not rank among the top predictors overall, they could enhance model performance when combined with primary risk factors. Future work should explore their weighted contributions in stratified analyses or their utility as interaction terms in subgroup-specific models.

Currently, there is a lack of dedicated predictive tools specifically targeting lower limb deep vein thrombosis (LDVT) following colorectal cancer surgery. Traditional models such as the Caprini score and the CRC-VTE model (AUC = 0.786) (42) are based on conventional logistic regression approaches. These models rely on predefined variables and linear assumptions, which limit their ability to fully capture potential nonlinear relationships and interactions among variables, thereby reducing their adaptability to complex clinical scenarios.

In contrast, machine learning (ML) techniques are well-suited for handling high-dimensional data and identifying complex nonlinear relationships and interactions among variables. In this study, we developed a CRC-LDVT prediction model using the Random Forest (RF) algorithm and applied SHAP analysis to interpret the model’s predictions. SHAP allowed us to quantify the contribution of each predictor clearly, highlighting key features such as D-dimer, preoperative bowel obstruction, and age. Importantly, the dynamic nature of D-dimer enables the model to capture real-time changes in thrombotic risk during the critical early postoperative period. Meanwhile, preoperative bowel obstruction—a factor specific to colorectal cancer patients—adds disease-specific information that substantially improves the model’s precision. This combination not only enhances the model’s predictive accuracy but also increases its transparency and clinical interpretability, effectively overcoming the common “black box” concerns associated with ML and promoting its practical application.

The primary limitation of this study lies in the single-source nature of the data, which was derived from patients at a tertiary hospital in China. This may restrict the generalizability of the model to other regions or populations. Additionally, although temporal validation was employed to assess the model’s stability over time, the lack of geographical validation could affect its applicability in different settings. Despite these limitations, the study successfully identified key risk factors for lower-extremity deep vein thrombosis (LDVT) following colorectal cancer surgery and developed the CRC-LDVT risk prediction model. These findings provide a solid foundation for future research and clinical applications. Future studies should aim to validate this model in multicenter cohorts, and explore real-time integration into clinical decision support systems.”

## 5 Conclusion

This study successfully developed the CRC-LDVT model for predicting lower-extremity deep vein thrombosis (LDVT) in patients following colorectal cancer surgery. Compared to traditional models, this model achieved an AUC of 0.942 (95% CI: 0.926-0.958), an accuracy of 0.894, an F1-Score of 0.924, a sensitivity of 0.945, and a Brier Score of 0.089. Additionally, we utilized SHAP values to interpret the model and developed an online web calculator(https://crc-ldvt.shinyapps.io/RF-model/).

## Data Availability

The original contributions presented in the study are included in the article/**Supplementary Material**. Further inquiries can be directed to the corresponding authors.
