# Supplementary material for: Machine learning-based prediction model and web calculator for postoperative LDVT in colorectal cancer
Source: Front Oncol. 2025 Oct 10;15:1673705. doi: 10.3389/fonc.2025.1673705 (PMC12549305; doi:10.3389/fonc.2025.1673705)

**Supplementary Materials**

Supplementary Table S1 List of Variables

| Category | Variables |
| --- | --- |
| Patient Baseline Characteristics | Age, sex, body mass index (BMI), history of smoking and alcohol consumption, ABO blood type |
| Past Medical History | History and grade of hypertension, history of diabetes, history of coronary heart disease and arrhythmia, chronic pulmonary diseases (including COPD), preoperative hematochezia/melena, intestinal obstruction, previous abdominal surgery, previous lower limb surgery, history of lower limb varicose veins |
| Surgical-Related Information | Preoperative bowel preparation (oral laxatives or enema), type of surgery (open or laparoscopic), intraoperative position, operative time, intraoperative blood loss, perioperative blood transfusion, length of preoperative hospital stay, number of catheters on the first preoperative day, postoperative urinary catheter/gastric tube and duration, postoperative ICU admission and length of stay, stoma formation, electrolyte disorders, infection, Caprini score, chemotherapy, incision fat liquefaction, placement of central venous catheter (CVC) |
| First Postoperative Peripheral Blood Tests | (1) Routine blood tests: RBC, HGB, mean hemoglobin concentration (g/l), WBC, neutrophils (NEU), lymphocytes (LYM), platelet count (PLT) (2) Coagulation tests: APTT, PT, D-dimer, fibrinogen (FIB) (3) Biochemical indicators: total protein, albumin, prealbumin (PA), total cholesterol, triglycerides, HDL, LDL, fasting blood glucose |
| Tumor Pathological Features | Pathological type, vascular invasion, carcinoembryonic antigen (CEA), postoperative pathological staging (according to the 9th edition TNM staging system, 2023) |

Supplementary Table S2. Hyperparameter Tuning Parameters and Values for ML Models

| Model | Hyperparameter | Description (English) | Values / Range |
| --- | --- | --- | --- |
| Random Forest | ntree | Number of trees | 100, 200, 300 |
|  | mtry | Number of variables randomly sampled at each split | 2, 4, 6 |
|  | maxnodes | Maximum number of terminal nodes | 10, 20, 30 |
| XGBoost | nrounds | Number of boosting rounds | 100, 200, 300 |
|  | max_depth | Maximum depth of a tree | 3, 5, 7 |
|  | eta | Learning rate | 0.01, 0.1, 0.3 |
|  | gamma | Minimum loss reduction to make a split | 0, 1, 5 |
| LightGBM | num_iterations | Number of boosting iterations | 100, 200, 300 |
|  | num_leaves | Maximum number of leaves in one tree | 31, 50, 70 |
|  | learning_rate | Learning rate | 0.01, 0.1, 0.2 |
| Support Vector Machine (SVM) | cost | Regularization parameter | 0.1, 1, 10 |
|  | gamma | Kernel coefficient (for RBF kernel) | 0.01, 0.1, 1 |
| Logistic Regression (glmnet) | alpha | Mixing parameter (0=ridge, 1=lasso) | 0, 0.5, 1 |
|  | lambda | Regularization strength | 0.01, 0.1, 1 |
| Decision Tree (rpart) | maxdepth | Maximum depth of the tree | 5, 10, 20 |
|  | minsplit | Minimum number of samples to split an internal node | 2, 5, 10 |
| Multilayer Perceptron (nnet) | size | Number of units in the hidden layer | 5, 10, 20 |
|  | decay | Weight decay parameter (L2 regularization) | 0.0001, 0.001, 0.01 |
|  | maxit | Maximum number of iterations | 100, 200, 300 |
| K-Nearest Neighbors (class) | k | Number of neighbors | 3, 5, 7 |

Supplementary Table S3 Top 10 Predictive Variables Ranked by Importance in Various ML Models

| Variable Rank | Logistic | SVM | RF | XGBoost | LightGBM | MLP | KNN | DT |
| --- | --- | --- | --- | --- | --- | --- | --- | --- |
| 1 | D-dimer | D-dimer | D-dimer | D-dimer | D-dimer | D-dimer | D-dimer | D-dimer |
| 2 | Intraoperative blood loss | Intraoperative blood loss | Ileus | Ileus | Ileus | Age | Ileus | Age |
| 3 | Age | Age | Age | Age | Caprini score | Number of catheters on postoperative day 1 | Age | Intraoperative blood loss |
| 4 | Number of catheters on postoperative day 1 | Number of catheters on postoperative day 1 | Caprini score | Arrhythmia | Ureter time | Ileus | Caprini score | Ileus |
| 5 | Varicosity | Varicosity | Infection | Infection | Age | Intraoperative blood loss | Infection | Number of catheters on postoperative day 1 |
| 6 | Ileus | Ileus | Arrhythmia | Diabetes | Arrhythmia | Arrhythmia | Arrhythmia | Caprini score |
| 7 | Ureter time | Ureter time | Diabetes | Caprini score | Infection | Varicosity | History of diabetes | Ureter time |
| 8 | Arrhythmia | Arrhythmia | Varicosity | Number of catheters on postoperative day 1 | Intraoperative blood loss | Infection | Varicosity | Nasogastric tube placement |
| 9 | History of alcohol use | History of alcohol use | Ureter time | Ureter time | Diabetes | Ureter time | Ureter time | Arrhythmia |
| 10 | Infection | Infection | Intraoperative blood loss | Intraoperative blood loss | Number of catheters on postoperative day 1 | Caprini score | Intraoperative blood loss | Coronary heart disease |

Supplementary Table S4 Performance Evaluation of Each Variable Model in the Validation Cohort

| **model** | **AUC (95% CI)** | **accuracy** | **sensitivity** | **specificity** | **PPV** | **NPV** | **F1_score** | **J_index** | **Brier Score** | **Balanced Accuracy** |
| --- | --- | --- | --- | --- | --- | --- | --- | --- | --- | --- |
| Variable = 8 | 0.862 (0.811–0.9138) | 0.76 | 0.74 | 0.815 | 0.915 | 0.537 | 0.818 | 0.555 | 0.1399 | 0.777 |
| Variable = 9 | 0.865 (0.8135–0.9165) | 0.75 | 0.699 | 0.889 | 0.944 | 0.522 | 0.803 | 0.588 | 0.1367 | 0.794 |
| Variable = 10 | 0.897（0.848-0.946） | 0.805 | 0.815 | 0.778 | 0.908 | 0.609 | 0.859 | 0.593 | 0.115 | 0.796 |
| Variable = 11 | 0.885 (0.8321–0.9372) | 0.765 | 0.719 | 0.889 | 0.946 | 0.539 | 0.817 | 0.608 | 0.1237 | 0.804 |
| Variable = 13 | 0.884 (0.8284–0.9392) | 0.765 | 0.719 | 0.889 | 0.946 | 0.539 | 0.817 | 0.608 | 0.1199 | 0.804 |

Supplementary Fig S1 Sample Screening Flowchart


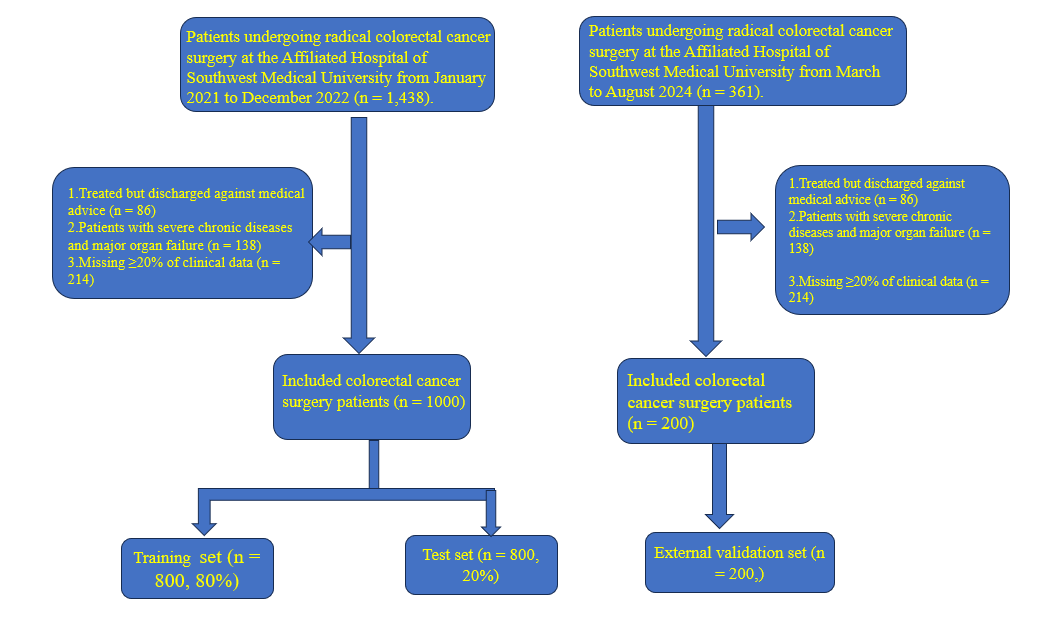

Supplement: Supplementary file 1 [file DataSheet1.docx]
